# An Association Study of the Signal Transducer and Activator of Transcription 6 Gene With Periodic Psychosis

**DOI:** 10.4306/pi.2008.5.1.41

**Published:** 2008-03-31

**Authors:** Seiya Kawashige, Tetsufumi Kanazawa, Atsushi Tsutsumi, Hiroki Kikuyama, Hiroyuki Uenishi, Jun Koh, Hiroshi Yoneda

**Affiliations:** 1 Department of Neuropsychiatry, Osaka Medical College, Takatsuki, Osaka, Japan.; 2 Course of Letters, Kansai University, Suita, Osaka, Japan.

**Keywords:** Signal transducer and activator of transcription 6, Genetic association study, Periodic psychosis, Atypical psychosis, Haplotypes

## Abstract

**Objective:**

Recent molecular and genetic investigations have suggested that the current nosology for major psychiatric disorders, based on the "two-entities-principal" is not accurate with respect to clinical observations; patient groups that do not fit to the current operative diagnostic boundaries are readily identified. We aimed to perform an investigation of the signal transducer and activator of transcription 6 (STAT6) gene (located on 12q13), which has an important role in the apoptotic cascade, with patients suffering from periodic psychosis.

**Methods:**

Genetic association study has been employed for the current work. Investigated six tag-SNPs were chosen from Hapmap database.

**Results:**

Among six tag-SNPs, one marker (rs10783813), located in the STAT6 gene, showed modest association (p<0.05), although no marker or haplotype block showed association after Bonferroni's correction.

**Conclusion:**

Future studies will reveal the etiological role of STAT6, and of other genes of the apoptotic cascade, in major psychiatric disorders.

## Introduction

Recent molecular genetic investigations of major psychiatric disorders have resulted in skepticism of the accuracy of the "two-entities-principle". The two-entitie-sprincipal postulates that major psychiatric disorders are divided into two groups, namely schizophrenia and bipolar disorder. This nosology was proposed by Kraepelin^1^,^2^ in the 19^th^ century and, despite criticism, it has survived through the centuries and still influences current operational diagnostics, such as the International Classification of Disease (ICD)^3^ for the Diagnostic and Statistical Manual of Mental Disorders (DSM).^4^ These classification systems have reduced the confusion between clinicians tra ined under different schools and their use has, therefore, become widespread all over the world. Although these classifications have been updated several times, the concept of Kraepelin's dichotomy still remains. However it has been difficult to clinically divide patients. Even in the field of molecular genetics there are several genes, such as Neureglin 1 (NRG1)^5^,^6^ and Disrupted in Schizophrenia 1 (DISC1),^7^,^8^ that are candidates for both bipolar disorder and for schizophrenia. This accumulated evidence suggests that the current nosology is not appropriate for precisely separating the major psychiatric disorders, nor for investigating candidate gene(s). Before the concerns of these operative criteria, schools named the patient group suffering from periodic psychosis as bouffés délirantes (French), reactive psychose (Northern Europe), schizoaffective psychosis (North America), psychogene Geisteskrankheitsformen (Denmark), zykloide Psychosis (German), and atypical psychosis (Japan). Although there were differences among these classifications, the past advocates labeled patients with this pathology due a better prognosis compared to those with common psychosis. This clinical insight is important with respect to treatment, while accurate taxonomy is essential to determine precise etiology. In the current study, using an association method, we aimed to determine genetic factors that are involved in this periodic psychosis. The DNA samples recruited were grouped according to three relatively solid criteria: 1) In a longitudinal time course, psychotic symptoms occur episodically, with partial or full remission, 2) Onset of prominent psychotic symptoms within 4 weeks of the first noticeable change from usual behavior or functioning, 3) Confusion of perplexity at the height of the psychotic episode (The description of 1) is derived from schizophrenia in DSM-IV. 2) and 3) are derived from schizophreniform disorder with good prognostic features). Sixty-nine samples were collected in the western area of Japan under these criteria.

Recent neuroimaging studies have identified low volumes of cortical gray matter in schizophrenia and suggested that apoptosis (programmed cell death) might cause this change.^9^,^10^ Apoptosis induces the up-regulation of prostaglandin E2 (PGE2), which suppresses T helper cell 1 (Th1) cytokine, resulting in a status known as the Th2 shift. Avgustin et al. reported a Th2 shift (Th2 > Th1) in patients with a relapse of schizophrenic symptoms.^11^ The Th2 shift increases mRNA levels of interleukin-4, which is a main cytokine of Th2. Interleukin-4 subsequently induces the phosphorylated signal transducer and activator of transcription 6, (STAT6) (locating on 12q 13). STAT6 is a transcriptional activator of Bcl-XL, which is a potent inhibitor of apoptosis. STAT6-deficient mice demonstrate the down-regulation of striatal dopamine transporter (DAT), weaker Prepulse Inhibition (PPI) and, in addition, they become hyperactive.^12^,^13^ Therefore, the absence of STAT6 results in the alteration of dopamine levels and of apoptosis in the brain. It is suspected that the progressive volume decrease of gray matter in schizophrenia is caused by the disturbance of this cascade. In periodic psychosis, continuous decline of social level is seldom seen, so it is not expected that significant apoptosis occurs in such patients. In schizophrenic brain, showing progressive recession, we hypothesized that STAT6, which is located down-stream in the apoptotic cascade, might be a cause of periodic psychosis. In addition, acute symptoms of periodic psychosis include an inflammatory response that is reversible with treatment by antipsychotic drugs. Therefore, we aimed to reveal the relationship between STAT 6 and periodic psychosis using the method of genetic association.

## Methods

### Subjects

Sixty-nine periodic psychosis subjects were recruited, while 123 normal controls were investigated. Two or more psychiatrists reviewed all of the recruited participants, who are ethnically Japanese. Informed consent was obtained from each subject.

### Single nucleotide polymorphism selection

Six single nucleotide polymorphisms (SNPs) were selected according to the following criteria;

1) Suggested as tag SNP by Hapmap data (Data Released #22, dbSNP b126).

2) Minor allele frequency is greater than 0.05 in a Japanese population. The detail of the six selected SNPs, primers and probes are shown in Table 1. Genotyping was performed by the fluorescence resonance energy transfer method (FRET) using the Light Cycler System (Roche). Statistical analysis was performed by SPSS software (ver 15.0) and by Haploview (ver 4.0).^14^

### Ethical consideration

The current study was approved by the ethical committee of Osaka Medical College, Japan.

## Results

**FIGURE 1:**
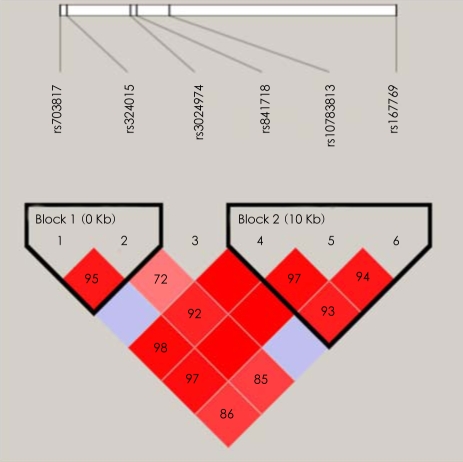
Linkage disequilibrium block structure (high D' block is shown in deep red).

**FIGURE 2:**
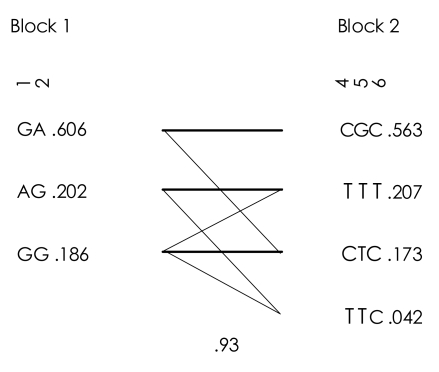
Linkage disequilibrium haplotypes.

One single SNP (rs10783813) showed a significant association (p=0.0458, not corrected), although it did not reach significance level after Bonferroni's correction (Table 1). In addition, several markers, such as rs841718 and rs167769, indicated the tendency to be associated with periodic psychosis. After the testing by Haploview, two blocks were constructed (Figure 1). Only one LD-block haplotypes (T-T-C in Block 2, Figure 2) indicated an association with the illness of interest in this research (p=0.042), while this pattern did not survive after the multiple-test correction.

## Discussion

The limitation of this study was the relatively smaller number of subjects chosen, due to the solid criteria for periodic psychosis adopted. Of the SNPs analyzed we found only a tendency for association. None of the single markers showed association after Bonferroni's Correction, while no haplotype showed significance of less than 0.05. Therefore, we are only able to state that STAT6 has a tendency to be associated with periodic psychosis. One further concern is not to be able to demonstrate the SNP association data of schizophrenia itself; clearer conclusions could be drawn if we could compare our data with that for schizophrenia.

As described before, the current nosology for major psychiatric disorders has been influenced by the hypothesis proposed by Kraepelin. Mitsuda of Osaka Medical College, proposed the concept named "atypical psychosis" in 1941. This concept is characterized by better long-term prognosis and confusional state with acute onset. The prevalence in females is generally greater than in males. Although this concept not adopted in the recent classification systems, it is still easy to find patients whose diagnosis lies across the two major psychoses. So far, several studies on the endophenotypes, detected by CT^15^ or by MRI,^16^ have shown better structural alteration in the brain of "atypical psychosis" suffers compared to that in schizophrenic brain. Moreover, the present study found a tendency of STAT6 to be associated with "atypical psychosis". Periodic onset of psychosis is regarded as part of the course of schizophrenia, however, the possibility of "atypical psychosis" must be ruled out in order to reduce the unnecessary use of antipsychotic agents. The current investigation may potentially explain the mechanism of periodic onset of psychosis, although future work will be needed to reveal the etiological role of STAT6 at the molecular level.

## Figures and Tables

**TABLE 1:**
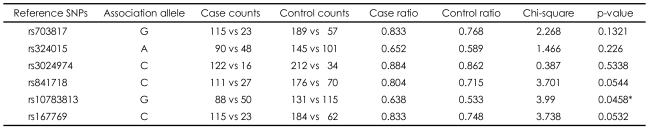
Results of single nucleotide polymorphisms (SNPs)

^*^Modest significance without multiple-test correction
